# Impact of the Stool-Based Xpert Test on Childhood Tuberculosis Diagnosis in Selected States in Nigeria

**DOI:** 10.3390/tropicalmed9050100

**Published:** 2024-05-01

**Authors:** Nkiru Nwokoye, Bethrand Odume, Peter Nwadike, Ikechukwu Anaedobe, Zirra Mangoro, Michael Umoren, Chidubem Ogbudebe, Ogoamaka Chukwuogo, Sani Useni, Debby Nongo, Rupert Eneogu, Emeka Elom, Petra De Haas, Mustapha Gidado

**Affiliations:** 1 Technical Department, KNCV Nigeria, Abuja 900103, Nigeria; bodume@kncvnigeria.org (B.O.); pnwadike@kncvnigeria.org (P.N.); ianaedobe@kncvnigeria.org (I.A.); zmangoro@kncvnigeria.org (Z.M.); mumoren@kncvnigeria.org (M.U.); cogbudebe@kncvnigeria.org (C.O.); ochukwuogo@kncvnigeria.org (O.C.); suseni@kncvnigeria.org (S.U.); 2 Program Management Department, HIV/AIDS & TB Office, USAID, Abuja 900103, Nigeria; dnongo@usaid.gov (D.N.); reneogu@usaid.gov (R.E.); 3 The Programs and Coordination Department, National Tuberculosis, Leprosy & Buruli Ulcer Control Program, Abuja 904101, Nigeria; elomek@yahoo.com; 4 Technical Department, KNCV Tuberculosis Foundation, 2516 AB The Hague, The Netherlands; petra.dehaas@kncvtbc.org (P.D.H.); mustapha.gidado@kncvtbc.org (M.G.)

**Keywords:** tuberculosis, stool, Xpert MTB/RIF, childhood TB

## Abstract

Background: In Nigeria, most children with tuberculosis (TB) present at primary health clinics where there are limited personnel skilled in collecting appropriate respiratory specimens from those who cannot produce sputum. KNCV Nigeria, in collaboration with the National Tuberculosis Control Program, implemented a modified simple, one-step (SOS), stool-based Xpert MTB/RIF method for diagnosis of TB in children who cannot expectorate sputum. We evaluated the impact of its implementation on childhood TB diagnosis. Method: A cross-sectional study was conducted across 14 selected states using secondary data of children presumed to have TB. Stool was collected from children presumed to have TB and processed using Xpert. Result: Out of 52,117 presumptive TB cases, 52% were male and 59.7% were under 5 years old. A total of 2440 (5%) cases were diagnosed with TB, and 2307 (95%) were placed on treatment. Annual TB notifications increased significantly after the introduction of the stool-based Xpert test when compared to those in the pre-implementation period. Increasing contributions from stool testing were observed throughout the implementation period, except in 2020 during the COVID-19 era. Overall, stool Xpert testing improved childhood TB notification in the studied states. Interventions aimed at awareness creation, capacity building, and active case finding improved the performance of the test.

## 1. Introduction

The World Health Organization (WHO) estimated that children accounted for 4.6% and 14% of TB-related mortality globally in 2011 and 2021, respectively [1]. Over half a million new cases of childhood TB occur every year, with most deaths occurring in children below five years of age and before the initiation of treatment [2]. The burden of TB in children is mainly due to undiagnosed TB and late diagnosis of adult TB, which creates a reservoir for transmission to children. TB can rapidly progress in children because of their immature immune systems [3,4]. Adolescents are often bacilli-ferrous and may transmit TB, develop excavated pulmonary forms, and cough and expectorate sputum, similarly to adults [5]. Clinical and radiological findings such as hemoptysis, cough, cavities, and upper-lobe consolidation are generally reliable and well-established in adults. In contrast, these findings might be more challenging to evaluate in children due to nonspecific symptoms and heterogeneous radiological findings [6]. High suspicion and rapid detection of TB in children should enable more rapid treatment to be initiated, leading to improved outcomes [2]. A bacteriological diagnosis reduces the risk of misdiagnosis, especially for drug-resistant TB, and prompts the initiation of effective treatment [2]. Obtaining spontaneously expectorated sputum represents a significant challenge for timely TB diagnosis in young children, necessitating the use of respiratory specimens such as gastric aspirates, induced sputum, nasopharyngeal aspirates, and bronchoalveolar lavage, which require resources and invasive procedures to collect [7].

In Nigeria, just like in other parts of the world, most children with TB present first at a primary health clinic, where there are limited skilled personnel and necessary equipment to collect appropriate respiratory specimens for diagnosis [7,8,9]. In such settings, TB diagnosis in children is primarily performed using sputum specimens, leading to a high proportion of missed cases and persistent poor case notification in children. Furthermore, sputum from children is often paucibacillary, as children are less likely to form cavitary lesions in the lungs to contain the TB bacilli [10]. Consequently, with sputum testing, most children with TB remain undetected [11]. Since 2020, stool specimens have been recommended by the World Health Organization (WHO) for TB diagnosis in children [12,13]. National TB programs are increasingly adopting this recommendation. Studies have reported the sensitivity of stool testing to be 50–67% and the specificity to be over 98–99% [14,15].

In Nigeria, however, stool testing is limited to TB reference laboratories because the centrifugation step has biosafety implications when performed outside of a containment lab. KNCV Nigeria, in collaboration with the National Tuberculosis, Leprosy, and Buruli Ulcer Control Program of Nigeria, deployed the simple, one-step (SOS) stool-based Xpert testing [7] for TB diagnosis in children who could not expectorate sputum of sufficient quality and quantity. The SOS stool processing method is recommended by global laboratory initiatives and the WHO [3,16]. This method is easily and widely implemented in all health facilities with functional Xpert machines, including the peripheral level [17]. It requires low-to-moderate biosafety, similar to the Xpert technique, and has proven to be a robust method [18]. The implementation of stool-based testing will significantly impact the number of children obtaining bacteriological TB confirmation, as well as the rifampicin resistance profile. As a result, more childhood TB cases will be diagnosed at lower levels of the healthcare system, with reduced costs for both the system and families [14]. The decentralization of stool-based Xpert testing in Nigeria is expected to expand access to bacteriological diagnosis of childhood TB. We aim to evaluate the impact of stool-based Xpert test implementation on childhood TB diagnosis in selected states.

## 2. Materials and Methods

### 2.1. Study Design

The study design was cross-sectional, using secondary data from 52,117 children presumed to have TB selected from 1082 health facilities across 14 states.

### 2.2. Setting

The study was conducted in Nigeria, a culturally diverse federation of 36 states and the Federal Capital Territory. It is a country located in West Africa with an estimated population of 202 million people and a Gross Domestic Product (GDP) growth rate of 3.46% in the fourth quarter of 2023. Nigeria has the sixth greatest burden of TB in the world and the greatest in Africa. Out of 479,000 TB cases estimated by the WHO in 2022, Nigeria notified 285,561. Seven percent of the cases were children aged 0–14 years.

**Figure 1:**
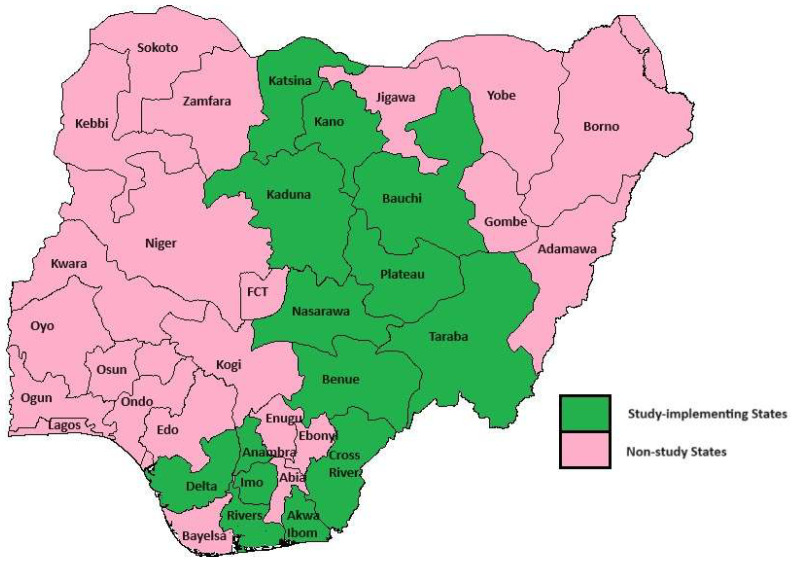
A map of Nigeria showing the study states.

A total of 1082 health facilities (11 tertiary, 126 secondary, and 945 primary) across 14 states were involved in the study. The 14 states are locations where KNCV Nigeria implements the USAID-funded TB Local Organization Network (LON) 1&2 project. The states are Anambra, Imo, Delta, Akwaibom, Rivers, Cross River, Nasarawa, Benue, Plateau, Taraba, Kano, Kaduna, Katsina, and Bauchi (Figure 1).

### 2.3. Intervention

The persistent low TB notification rate necessitated a national high-level meeting of TB stakeholders in 2020 to elucidate the challenges affecting low-bacteriological TB diagnosis in children and offer improvement solutions. A critical conclusion of the meeting was the need to decentralize the stool-based test to peripheral sites where most children seek help. A review of existing stool-based methods was conducted to identify the challenges associated with each procedure and the possible procedural steps that could be modified for better performance. By Q3, 2020, the simple, one-step, stool-based Xpert method was modified by introducing hard-formed stool emulsification with saline, along with a filtration step to eliminate debris that may inhibit the PCR process. 

The modified method was successfully verified by in-country TB experts and adopted for use in Nigeria. Consequently, the national TB guidelines and laboratory SOPs were reviewed to reflect the new stool-based procedure and introduced into routine testing in 14 KNCV-supported states. At the onset of implementation in Q4, 2020, a nationwide webinar was conducted to sensitize healthcare workers on the alternative specimen (stool) for TB diagnosis in children, which created demand for the test. In addition, laboratory staff were trained on the procedure. Furthermore, a YouTube demonstration video of the procedure was developed and disseminated widely to guide laboratory staff in need of further physical training and to serve as a refresher course for others. The video prompted a rapid scale-up of test availability to all states in the federation. Massive sensitization webinars for a combined total of 2838 healthcare workers held at the state level in Q3, 2022 significantly increased awareness of stool-based Xpert testing among the healthcare workers. The state TB programs, implementing partners, and other TB stakeholders collaboratively organized the sensitization. There was a rapid uptake of the stool test among clinicians, with increased referrals for testing. Nigeria organized a childhood TB testing week in Q2, 2023, to detect TB cases among children. It was characterized by massive sensitization of HCWs and the public through motorized campaigns, the airing of TB jingles, and the provision of laboratory commodities and human resources to support diagnostic investigation. Furthermore, GeneXpert laboratories were regularly optimized with solar systems and prompt equipment repairs to minimize service interruptions. 

### 2.4. Study Population and Specimen Collection

All children less than 15 years of age presenting across 1082 health facilities with pulmonary TB symptoms of cough >2 weeks, weight loss, appetite loss, persistent fever without an apparent cause, night sweats, or history of close contact with a TB patient [19] were requested to submit a sputum specimen. Those who could not produce sputum were encouraged to submit a stool specimen instead. No child submitted both sputum and stool specimens. The specimens were transported immediately in a cold chain to the nearest GeneXpert laboratory for processing. 

### 2.5. Procedure for Stool Processing

Briefly, 2 mL of saline was added to about 1 g of hard-formed stool to emulsify it into a liquid slurry. This step was bypassed if the stool was soft or watery. The stool saline mixture was allowed to stand for 15 min for dissolution. Approximately 6 mL of sample reagent was added to the mixture to make a 3:1 dilution that was incubated for 15 min. The supernatant was filtered into a well-labeled specimen container (to separate debris) and added into an Xpert ultra cartridge for GeneXpert testing. A bacteriological diagnosis of TB was made with a positive GeneXpert result. Both bacteriologically confirmed and clinically positive patients were placed on treatment. 

### 2.6. Data Variables, Sources, and Collection

An electronic database of children presumed to have TB registered from October 2020 to September 2023 in the 1082 health facilities across the 14 KNCV-supported states was reviewed. De-identified summary data were extracted into an MS Excel-based data collection form designed for the study. This included age, sex, specimen collection status, specimen type, TB testing, TB diagnosis, and treatment status. Information on childhood TB notified from 2017 to 2020 was obtained from the state quarterly TB statistical report and verified using the facility TB Register.

### 2.7. Statistical Analysis

Data analysis was conducted using SPSS version 20 and other statistical software as appropriate. Descriptive statistics were performed to determine the testing yield for the different specimen methods. Results are presented appropriately in tables and charts using frequencies and percentages.

### 2.8. Ethical Considerations

No direct contact with human subjects was made; only de-identified pooled program data that formed part of the standard of care were used, and informed consent was not required.

## 3. Results

The demographic information of the study group who submitted sputum for evaluation is shown in Table 1. Of the 391,217 sputum specimens sent to the GeneXpert laboratory, 29.5% were from children aged 0–4 years, and 70.5% were from children aged 5–14 years. Ninety-two percent of the sputum specimens sent to the GeneXpert lab were evaluated. Of those evaluated, 12,593 (3.5%) were diagnosed with TB. About 1% of the total diagnosed TB cases were DR-TB, while 95.2% of the total TB cases were started on treatment.

Table 2 shows the demographic information of the study group who submitted stool samples. Of the 52,117 stool specimens sent to the GeneXpert laboratory, 59.7% were from individuals aged 0–4 years, and 40.3% were from those aged 5–14 years. The majority (94.6%) of stool specimens sent to the GeneXpert laboratory were evaluated. Among those evaluated, 2440 (4.8%) were diagnosed with TB. About 1.1% of the total TB cases diagnosed were DR-TB, and 94.6% of the total TB cases were started on treatment.

**Figure 2:**
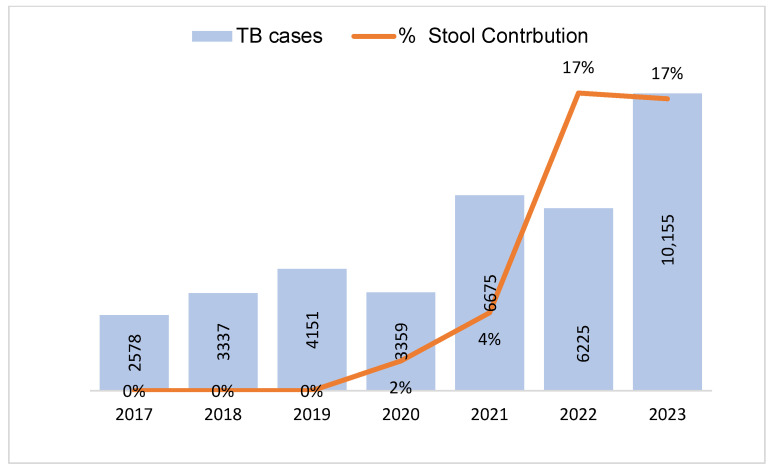
Annual trend of diagnosed TB cases and stool testing contribution in children aged 0–14 years in 14 states of Nigeria between 2017 and 2023.

The yearly trends of detected TB cases and the percentage contribution of stool testing are shown in Figure 2. Increases in the annual number of TB cases and percentage contribution of stool testing were observed throughout the implementation of stool GeneXpert testing, except in the 2020 COVID-19 era. The highest contribution, 17%, was recorded in 2022 and 2023, while the lowest, 2%, was recorded in 2020.

Table 3 depicts the clients’ demographic and clinical characteristics by age group and gender. Children in the 0–4 age group were mainly referred (59.7%) and evaluated (59.9%) for stool tests. The number of diagnosed DS-TB cases was higher within the 0–4 age group (58.1%). Among these younger children, males were predominantly referred (31.7%) for stool testing and diagnosed with DS-TB (32.4%). The number placed on treatment was also higher in 0–4-year-old boys (32.7%). Children in the 5–14-year-old group, on the other hand, had a higher proportion (53.8%) of DR-TB cases, mainly seen among the male gender (64.3%).

**Figure 3:**
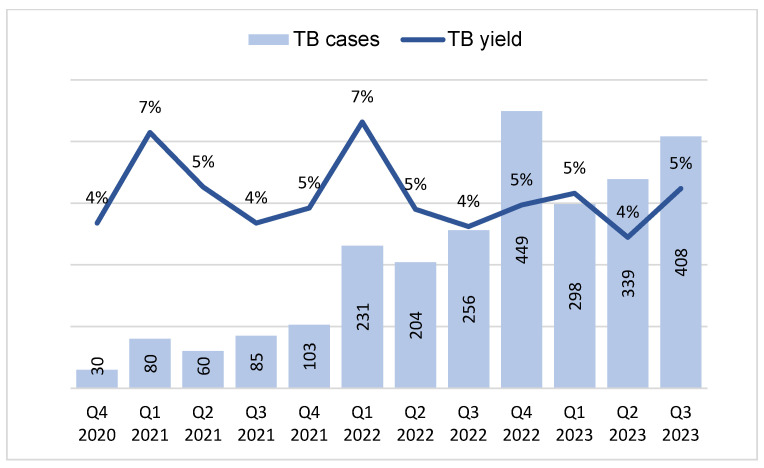
Quarterly trend of bacteriologically diagnosed TB cases and TB yield from stool tests in children aged 0–14 years in 14 states of Nigeria between quarter 1, 2010, and quarter 3, 2023.

The quarterly trends of TB diagnosis and yield are presented in Figure 3. The highest yield of 7% was recorded in Q1, 2021, and Q1, 2022, while the highest number of cases was recorded in Q4, 2022.

## 4. Discussion

The upward trajectory in childhood TB notification after the introduction of stool-based Xpert testing in 2020 demonstrates the pivotal role of this intervention and other targeted interventions in improving childhood TB notification across the 14 states. Poor notification levels in 2019 and 2020 prompted accelerated efforts to tackle this issue. Interventions (such as the screening of malnourished children in nutritional and immunization clinics, active case identification in target populations like schools and orphanage homes, and contact investigation) that intentionally and actively identified children with presumptive TB and referred them for bacteriological diagnosis yielded positive outcomes. Unfortunately, although a greater number of these children were identified, most of them could not produce sputum. Most of these expectorated sputum specimens yielded negative Xpert results because they were of poor quality. This is evident from the proportion of TB cases 12593 (3.5%) derived from sputum evaluation after testing 361,452 specimens.

The introduction of stool as an alternative specimen for children who could not expectorate a quality specimen expanded access to bacteriological diagnosis. A TB yield of 4.8% from the stool-based test is very promising and higher than the 3.5% yield from the sputum-based test. The ease of collection of stool specimens and the low biosafety risk associated with laboratory procedures have decentralized testing to peripheral labs where underserved populations first seek help. Studies have shown that older children (5–14 years) should be able to expectorate quality sputum specimens because they have adult-type cavitations that facilitate spontaneous expectoration [20,21]. Thus, such children are encouraged by healthcare workers to submit sputum. This is evident from the high proportion of sputum referrals among the 5–14 age group and more stool referrals among younger children (0–4 years). Interestingly, we observed that older children in the 5–14 age group had a higher TB yield from stool testing than the younger children. Considering this finding, it will be interesting to evaluate the potential benefit of stool as an alternative specimen for TB diagnosis in presumptive adults, especially HIV patients who do not present with a cough.

In our study, TB was common among children aged 0–4 years. This was expected because children in high-burden settings present early with TB. They usually develop TB during the first year of infection. Hence, TB in this age group is an indication of recent and ongoing transmission of infection [11]. This agrees with previous studies, which reported a high proportion of childhood TB cases among the 0–4-year age group [21,22,23]. In contrast to our finding, a study that utilized sputum and microscopy method reported more TB cases among the 5–15 age group [24], suggesting that the 0–4 age group, the members of which usually find it challenging to produce sputum, may have been excluded from the beginning of the cascade, depriving them of opportunities for bacteriological examination. The majority that escaped exclusion via sputum collection may have been missed by microscopy due to the paucibacillary nature of the infection, which microscopy is not sensitive enough to detect.

A higher number of TB cases was observed among boys within the 0–4 age group. Sexual dimorphism in TB incidence has mainly been seen in adults. A study on TB epidemiology in children conducted across seven high-income countries reported gender differences in the incidence rates for tuberculosis, with higher rates in boys aged less than 1, no significant differences in boys aged 1–9, and higher rates in boys/men older than 15. A higher incidence in female children was observed in the 10–14 age group [22].

Approximately 5% of the total processed samples were positive for TB. This rate is comparable to but lower than a few studies that reported stool positivity rates. A study conducted in an Ethiopian teaching and referral hospital, where the majority (54%) of the study participants were severely malnourished children, recorded a 6.6% positivity rate [23]. Two other studies conducted in Indonesia reported stool positivity rates of 13.4% and 8.3%, respectively. These higher rates were mainly due to the targeted study population, encompassing children admitted with severe symptoms and a higher probability of pulmonary TB [25]. The second study’s sample size was small, with 36 stool samples [26]. TB screening in our study was conducted among the general population, which may have resulted in the 5% positivity rate. While this may be seen as a low yield compared to previous studies, the risk of morbidity and mortality and continuous transmission of infection in the community justified our active screening in health facilities and communities.

There was an annual rise in the number of diagnosed TB cases and the percentage contribution from the stool-based Xpert test, except in 2020 during the COVID-19 era. The TB cases resulting from the stool test were identified through the efforts of TB stakeholders, who supported active case-finding activities and testing. Stool testing was introduced across 14 Nigerian states via a nationwide webinar in Q4, 2020, in addition to awareness creation among healthcare professionals, including pediatricians, DOTS officers, laboratory staff, and clinicians. This resulted in an increased demand for stool testing, with a 167% increase in TB cases diagnosed in the succeeding quarter. It was also observed that laboratory training sessions conducted across the 14 implementing states in Q4, 2021, resulted in a 124% increase in TB cases in Q1, 2022. TB yield also increased from 5% to 7%, indicating an improvement in the efficiency and quality of the test.

Other improvement strategies that enhanced stool test performance included massive healthcare worker sensitization via state-organized webinars and the Childhood TB Testing Week, a week-long activity dedicated to identifying TB cases among children. This was characterized by motorized campaigns, the airing of TB jingles, and the engagement of ad hoc staff to support testing in high-burden laboratories. A 75% increase in TB cases was recorded in Q4, 2022, following the webinar conducted in Q3, 2022. Similarly, TB cases increased by 20% in Q3, 2023, following the Childhood TB Testing Week, which was conducted in the preceding quarter. Over 97% of the stool samples referred to laboratories were tested, highlighting the need for functional laboratories and adequate human resources to conduct Xpert testing. The improved functionality of GeneXpert laboratories in recent years can be attributed to the prompt repair and replacement of GeneXpert ancillary equipment, human resource support to laboratories, and an improved sample referral system.

## 5. Conclusions

Overall, stool-based Xpert testing significantly contributed to improved childhood TB diagnosis and notification in the 14 studied states. Interventions aimed at awareness creation, capacity building, and active TB case finding in children are some of the strategies that improved and sustained the performance and quality of the test over time.

## Figures and Tables

**Table 1:** Demographic and clinical information of children who submitted sputum specimens for evaluation.

Variable	0–4 Years *n* (%)	5–14 Years *n* (%)	Total
Presumptive TB sent for sputum evaluation	115,271 (29.5)	275,946 (70.5)	391,217
Presumptive cases evaluated for TB	104,809 (29.0)	256,643 (71.0)	361,452
Total TB cases diagnosed	3108 (24.7)	9485 (75.3)	12,593
Diagnosed with DS-TB	3093 (24.7)	9438 (75.3)	12,531
Diagnosed with DR-TB	15 (24.2)	47 (75.8)	62
Total TB patients started on treatment	2948 (24.6)	9042 (75.4)	11,990
DS-TB patients started on treatment	2939 (24.6)	9021 (75.4)	11,960
DR-TB patients started on treatment	9 (30.0)	21 (70.0)	30
Presumptive evaluation rate	90.9%	93.0%	92.4%
TB yield	3.0%	3.7%	3.5%
Treatment enrollment rate	94.9%	95.3%	95.2%

DS-TB = Drug-Sensitive Tuberculosis; DR-TB = Drug-Resistant Tuberculosis; presumptive evaluation rate = (presumptive cases evaluated for TB/presumptive TB cases sent for sputum evaluation); TB yield = (total diagnosed TB cases/presumptive cases evaluated for TB); treatment enrollment rate = (total TB patients started on treatment/total diagnosed TB cases).

**Table 2:** Demographic information of children who submitted stool specimens for evaluation.

Variable	0–4 Years *n* (%)	5–14 Years *n* (%)	Total
Presumptive TB sent for stool evaluation	31,131 (59.7)	20,986 (40.3)	52,117
Presumptive cases evaluated for TB	30,455 (60.0)	20,319 (40.0)	50,774
Total diagnosed TB cases	1414 (58.0)	1026 (42.0)	2440
Diagnosed with DS-TB	1402 (58.1)	1012 (41.9)	2414
Diagnosed with DR TB	12 (46.2)	14 (53.8)	26
Total TB patients started on treatment	1351 (58.6)	956 (41.4)	2307
DS-TB patients started on treatment	1341 (58.6)	946 (41.4)	2287
DR-TB patients started on treatment	10 (50.0)	10 (50.0)	20
Presumptive evaluation rate	97.8%	96.8%	97.4%
TB yield	4.6%	5.0%	4.8%
Treatment enrollment rate	95.5%	93.4%	94.6%

DS-TB = drug-sensitive tuberculosis; DR-TB = drug-resistant tuberculosis; presumptive evaluation rate = (presumptive cases evaluated for TB/presumptive TB cases sent for sputum evaluation); TB yield = (total diagnosed TB cases/presumptive cases evaluated for TB); treatment enrollment rate = (total TB patients started on treatment/total diagnosed TB cases).

**Table 3:** Demographic and clinical characteristics of children from 14 Nigerian states who submitted stool specimens.

Variable	Sub-Category	Male		Female		Total
		0–4 Years *n* (%)	5–14 Years *n* (%)	0–4 Years *n* (%)	5–14 Years *n* (%)	
Stool sample referred		16,504 (31.7)	10,539 (20.2)	14,627 (28.1)	10,447 (28.1)	52,117
Stool sample evaluated for TB		16,235 (32.0)	10,167 (20.0)	14,220 (28.0)	10,152 (20.0)	50,774
TB patients diagnosed	DS-TB patients	783 (32.4)	525 (21.7)	619 (25.6)	487 (20.2)	2414
	DR-TB patients	7 (26.9)	9 (34.6)	5 (19.2)	5 (19.2)	26
TB patients started on treatment	DS-TB patients	747 (32.7)	491 (21.5)	594 (26.0)	455 (19.9)	2287
	DR-TB patients	5 (25.0)	6 (30.0)	5 (25.0)	4 (20.0)	20

DS-TB = drug-sensitive tuberculosis; DR-TB = drug-resistant tuberculosis.

## Data Availability

The data presented in this study are available upon request from the corresponding author. The data are not publicly available due to privacy and ethical restrictions.
